# Systolic and Diastolic Blood Pressure Are Independent Risk Factors for Diabetic Retinopathy in Patients with Type 2 Diabetes

**DOI:** 10.3390/biomedicines11082242

**Published:** 2023-08-10

**Authors:** Tomislav Bulum, Martina Tomić, Romano Vrabec, Neva Brkljačić, Spomenka Ljubić

**Affiliations:** 1 Department of Diabetes, Vuk Vrhovac University Clinic for Diabetes, Endocrinology and Metabolic Diseases, Merkur University Hospital, 10000 Zagreb, Croatia; 2 Medical School, University of Zagreb,10000 Zagreb, Croatia; 3 Department of Ophthalmology, Vuk Vrhovac University Clinic for Diabetes, Endocrinology and Metabolic Diseases, Merkur University Hospital, 10000 Zagreb, Croatia; 4 Department of Cardiology, Vuk Vrhovac University Clinic for Diabetes, Endocrinology and Metabolic Diseases, Merkur University Hospital, 10000 Zagreb, Croatia

**Keywords:** type 2 diabetes, diabetic retinopathy, risk factors, systolic blood pressure, diastolic blood pressure

## Abstract

Background and aims: Diabetic retinopathy (DR) is a microvascular complication of diabetes and represents the leading cause of blindness in working-age adults. The aim of this study was to investigate the risk factors for DR in patients with type 2 diabetes (T2DM) with and without diabetic nephropathy (DN). Methods: A total of 160 patients with T2DM were included in the study. Photodocumented retinopathy status was determined according to the EURODIAB protocol. Renal function was determined using creatinine-based estimated glomerular filtration rate (eGFR) and albumin-to-creatinine ratio (ACR). Binary univariate and multiple logistic regression analyses were performed to determine the main predictors of DR. Results: The prevalence of DR in this studied sample was 46.3%. No significant correlation was observed between DR and age, body mass index, serum lipids, and renal function. Binary logistic regression analysis (no DR/DR) showed that longer diabetes duration (*p* = 0.008), poor glycemic control (HbA1c) (*p* = 0.008), higher systolic blood pressure (*p* = 0.001), and diastolic blood pressure (*p* = 0.003) were the main predictors of DR in patients with T2DM. However, the influence of systolic blood pressure (AOR = 1.06, *p* = 0.004) and diastolic blood pressure (AOR = 1.12, *p* = 0.007) on DR development remained significant even after adjustment for diabetes duration and HbA1c. Conclusions: Our results suggest that systolic and diastolic blood pressure are independent risk factors for DR in patients with T2DM.

## 1. Introduction

The dramatic increase in the prevalence of diabetes mellitus and its complications positions this metabolic disorder as the most common non-communicable disease worldwide. It is estimated that about 700 million people worldwide by 2045 will have diabetes [1]. Despite new therapeutic options, there has also been an increase in chronic complications of diabetes [2]. Diabetic retinopathy (DR) is one of the major chronic complications of diabetes and still represents the leading vision-threatening disease in working-age adults [3]. In prospective studies, hyperglycemia and hypertension have been documented as the most important modifiable risk factors for developing DR, along with the duration of diabetes as the most important non-modifiable risk factor [4,5,6,7]. Most patients (about 90%) have type 2 diabetes (T2DM), in which overweight and abdominal obesity plays a crucial role in developing insulin resistance and diabetes. Abdominal obesity and insulin resistance are underlying conditions in developing metabolic syndrome-associated disorders like hypertension and dyslipidemia, which are connected with microvascular complications of diabetes, including DR [8].

The two most common microvascular complications in patients with T2DM are DR and diabetic nephropathy (DN). Since these two microvascular complications share a similar pathogenesis, they are supposed to develop in parallel in patients with diabetes, and the presence and severity of DR are accompanied by the presence and severity of DN [9,10]. However, DR and DN are more closely related and simultaneously progress in patients with type 1 diabetes than in patients with T2DM [11]. The reason could be that at the time of diagnosis of type 1 diabetes, most patients had neither DR nor DN, and over time and with complications development, there are more substantial effects of risk factors associated with DN, like hypertension, on DR development [12]. In T2DM, there is a well-recognized association between DR and DN—DN without DR is rare, but DR without DN is common. DR is associated with an increased risk of DN in patients with T2DM, and the predictive value of DR on subsequent DN risk for T2DM patients is relatively lower [13].

In addition to high blood glucose, blood pressure is also a significant risk factor for the development of DR because high blood pressure is transferred into retinal circulation, leading to injury of retinal endothelial cells [14]. The results from the large and prospective United Kingdom Prospective Diabetes Study (UKPDS) suggest that the risk of DR is increased in patients with T2DM and higher systolic blood pressure (SBP) compared to those with normal blood pressure [15]. Similar outcomes were found in a meta-analysis that included over 100,000 participants, where a decrease in SBP by 10 mmHg reduced the risk of DR by 13% [16]. Generally, there is a high prevalence of hypertension in patients with T2DM because at the time of diagnosis, around 40% of them have high blood pressure, and with increased age and the longer duration of diabetes, the percentage increases—up to 60% in those aged 75. Even in those with SBP < 140 mmHg and diastolic blood pressure (DBP) < 90 mmHg, there is a significant association between SBP and DR after adjusting for known risk factors like hemoglobin A_1_c, diabetes duration, and gender [17]. We previously described in T2DM the connection between hypertension and cataract development and the association between systolic blood pressure and the development and progression of DR in patients with type 1 diabetes with normal renal function (normoalbuminuria and estimated glomerular filtration rate over 60 mL/min/1.73 m^2^) [18,19].

The aim of this study was to explore the prevalence of DR and DN and risk factors for DR in patients with T2DM with and without DN.

## 2. Methods

### 2.1. Study Design and Ethics Statement

Our study was cross-sectional and performed between 15 December 2020 and 15 March 2021 at the Department of Diabetes, Department of Ophthalmology, and Department of Cardiology. The study was conducted following the Declaration of Helsinki and approved by the Hospital’s Ethics Committee (protocol number 05/01-849, approval date: 16 September 2020). Before any procedures and inclusion in the study, patients received written and oral information about the study and finally signed informed consent. All patients in the study agreed that their data and image documents could be used for publication in scientific and professional journals.

### 2.2. Patients

The study included a total of 160 patients with T2DM attending all three Departments on the same day. Patients with illnesses affecting eye and renal function or blood pressure, inflammatory diseases, or acute infectious diseases, and patients who were pregnant or had other forms of eye diseases were not included in the study.

**Figure 1:**
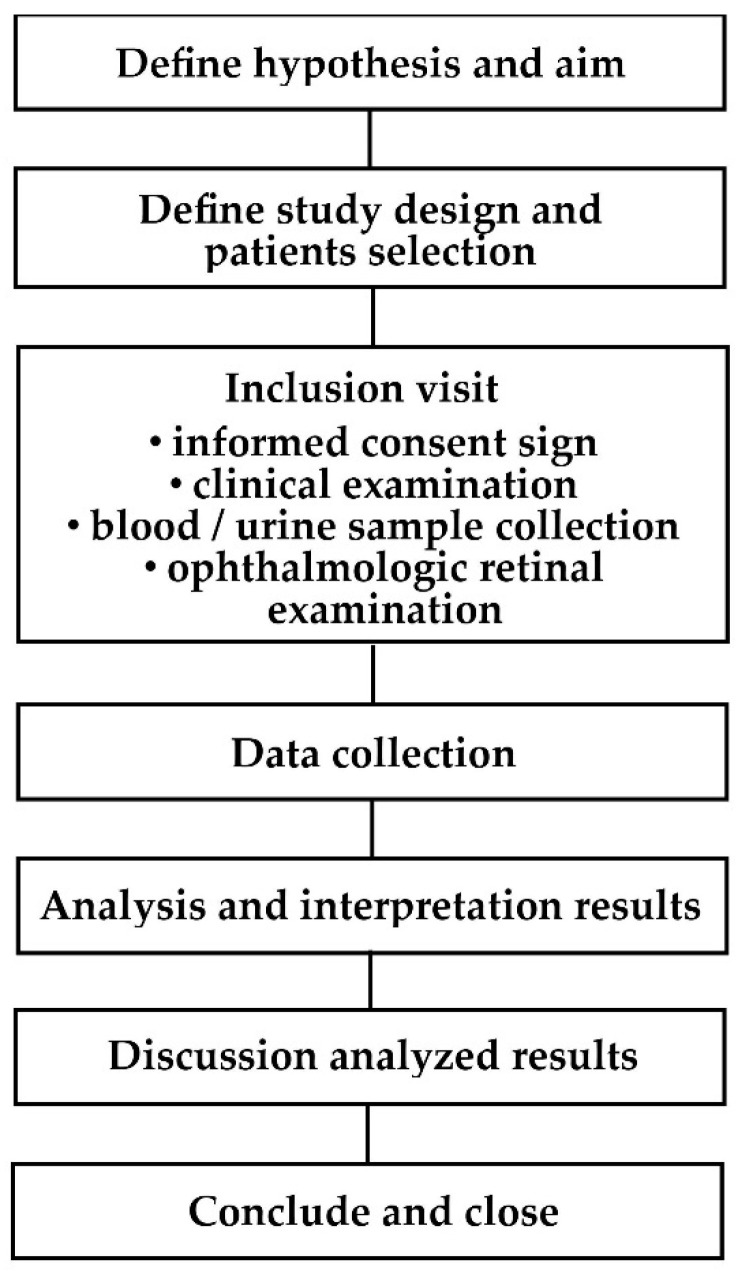
Design of experiment flow chart.

At the inclusion visit, complete clinical and ophthalmologic retinal examinations were performed in addition to blood and urine sample collection for laboratory analysis. Figure 1 presents the flow chart for designing the experiment.

### 2.3. Demographic Data and Clinical Characteristics

The demographic data of patients include age, gender, and diabetes duration. Weight and height were measured using a balance-beam scale and a wall-mounted stadiometer. Body mass index (BMI) was calculated by dividing weight and height squared (kg/m^2^). Systolic and diastolic blood pressure were measured by an ambulatory digital sphygmomanometer after a 10 min rest period, and a mean value of three measurements was used.

### 2.4. Markers of Glycemic Control and Lipid Metabolism

Fasting venous blood samples were collected in the morning after an overnight fast to determine metabolic risk factors: hemoglobin A_1_c (HbA_1c_), total cholesterol, HDL cholesterol, LDL cholesterol, and triglycerides. HbA_1_c was measured using an automated immunoturbidimetric procedure on a dedicated analyzer (Cobas Integra 400 plus, Roche Ltd., Basel, Switzerland), and serum lipids were determined using standard enzymatic methods on an automated analyzer (Beckman Coulter AU680, Beckman Coulter, Inc., Brea, CA, USA).

### 2.5. Indicators of Renal Function

Renal function was determined using serum creatinine-based glomerular filtration rate (GFR) and albumin-to-creatinine ratio (ACR). Serum creatinine was measured in a fasting blood sample using a routine laboratory method. GFR was estimated using the Chronic Kidney Disease Epidemiology Collaboration (CKD-EPI) formula [20], and a random urine sample was used to determine the ACR by turbidimetric immunoassay and photometric assays. Patients with an ACR < 3 mg/mmol were classified as normoalbuminuric, those with ACR ≥ 3 < 30 mg/mmol as microalbuminuric, while macroalbuminuria was classified as an ACR ≥ 30 mg/mmol. Chronic kidney disease/DN was defined as an estimated GFR < 60 mL/min/1.73 m^2^ and/or ACR ≥ 30 mg/mmol.

### 2.6. Ophthalmologic Retinal Examination

The ophthalmologic retinal examination included color fundus photography and optical coherence tomography (OCT) of the macula after mydriasis with eye drops containing 0.5% tropicamide. A conventional 45° fundus camera (Visucam, Zeiss) was used to obtain color fundus images following the EURODIAB (EUROpe and DIABetes) retinal photography methodology [21]. Using the EURODIAB criteria, two retina specialists (M.T., and R.V.) evaluated the images separately, and the final diagnosis for each patient was established based on the DR level of the poorer eye [21]. The macula was examined using Spectral Domain OCT (SD-OCT Copernicus REVO NX, Optopol Technology), and diabetic macular edema (DME) was assessed using the proposed international clinical diabetic retinopathy and diabetic macular edema disease severity ratings [22]. We excluded patients with DME from the study.

### 2.7. Statistical Analysis

Statistica™ software package version 14.0 (TIBCO Software Inc., Palo Alto, CA, USA) and SPSS software package version 23.0 (IBM, Armonk, NY, USA) were used for statistical analysis. After testing the normality of data distribution using the Kolmogorov–Smirnov test, descriptive results were expressed as means ± SD or median (min-max) for continuous variables and numbers (percentages) for categorical variables. For continuous data, differences between groups were tested by one-way ANOVA and Kruskal–Wallis tests. Scheffe’s post-hoc test and multiple comparisons of the Kruskal–Wallis test were used where needed. For categorical data testing, the Chi-square test was used. The Spearman’s rank correlation test was used to assess the presence of associations between examined variables. Binary univariate and multiple logistic regression analyses were performed to determine the main predictors of DR, while backward regression analysis was used to detect the main predictors of the ACR. The level of statistical significance was set at 0.05 in all analyses.

## 3. Results

### 3.1. Study Population

One hundred and sixty patients with T2DM (96 male/64 female) with a mean age of 64.3 ± 7.6 years and a mean diabetes duration of 14.0 ± 7.1 years were included in this cross-sectional study. Table 1 presents their basic and clinical characteristics, metabolic risk factors, and renal function. Except for elevated mean/median values of BMI (29.8 ± 4.7 kg/m^2^), SBP (135 (110–170) mmHg), HbA_1_c (7.1 (5.5–12.1)%), and LDL cholesterol (2.5 (0.9–7.1) mmol/L), the other analyzed variables—mean/median values of DBP (80 (70–110) mmHg), total cholesterol (4.6 (2.7–10.2) mmol/L), HDL cholesterol (1.3 (0.8–2.5) mmol/L), triglycerides (1.6 (0.5–7.0) mmol/L), serum creatinine (75.5 (42–163) µmol/L), estimated GFR (86.5 (32–108) mL/min/1.73 m^2^), and ACR (1.4 (0.3–49.6) mg/mmol)—were within the normal range for patients with T2DM. Corresponding to the medical records, 134 (83.7%) of patients received antihypertensive therapy and 128 (80%) received hypolipemic treatment, of which 108 (67.5%) took statins and 20 (12.5%) took fenofibrate.

According to the level of DR, patients were divided into groups: no DR (*n* = 86), mild/moderate nonproliferative DR (NPDR) (*n* = 44), and severe NPDR/proliferative DR (PDR) (*n* = 30). The three groups did not significantly differ in age, gender, and BMI, but a significant difference was observed in the diabetes duration (*p* = 0.017), SBP (*p* = 0.001), DBP (*p* = 0.003), and HbA_1_c (*p* = 0.005) (Table 2). Patients with severe NPDR/PDR had a longer diabetes duration (post-hoc Scheffe test, *p* = 0.027) and higher SBP (multiple comparisons, *p* < 0.001) than those with no DR. In contrast, no significant difference in diabetes duration and SBP was observed between the patients with mild/moderate NPDR and those with no DR. Multiple comparisons also showed a significant difference in DBP and HbA_1_c between the patients with severe NPDR/PDR and those with no DR (DBP, *p* = 0.017; HbA_1_c, *p* = 0.022), as well as patients with mild/moderate NPDR and those with no DR (DBP, *p* = 0.043; HbA_1_c, *p* = 0.030). The differences in DBP and HbA_1_c between the patients with severe NPDR/PDR and mild/moderate NPDR were not statistically significant. No significant differences in lipids and renal function were found between the groups according to the level of DR.

### 3.2. Prevalence of Microvascular Complications and Their Correlations

In our study cohort, the prevalence of DR was 46.3%. Of the 74 with DR, 44 (59.5%) had mild or moderate NPDR, and 30 (40.5%) had severe NPDR or PDR.

The prevalence of DN according to the eGFR category was 32.6%, though its prevalence according to the ACR classification was 12.5%. Of the 92 with eGFR ≤ 90 mL/min/1.73 m^2^, 62 (67.4%) had eGFR 60–89 mL/min/1.73 m^2^, while 30 (32.6%) had eGFR ≤ 59 mL/min/1.73 m^2^. Of the 32 with an ACR higher than 3 mg/mmol, an ACR of 3–30 mg/mmol was found in 28 (87.5%) patients, and an ACR > 30 mg/mmol only in 4 (12.5%) of them.

**Figure 2:**
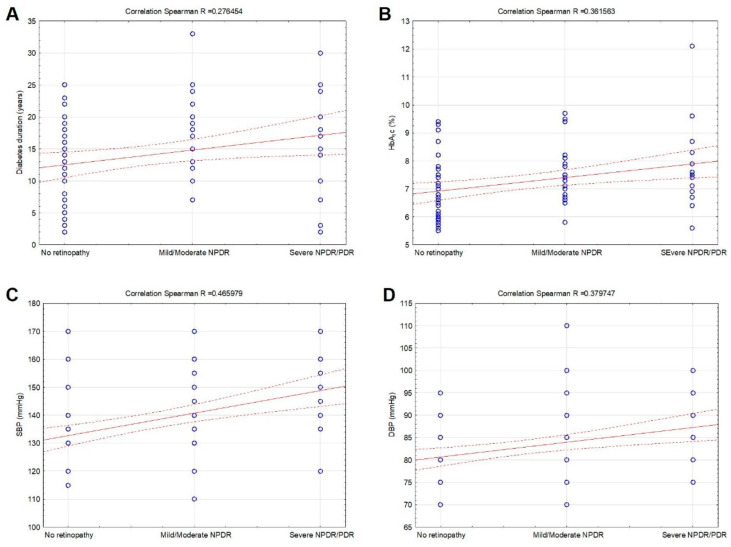
Correlations between diabetic retinopathy, diabetes duration (**A**), HbA_1_c (**B**), systolic blood pressure (SBP) (**C**), and diastolic blood pressure (DBP) (**D**) in patients with T2DM included in the study. Legend of the correlation linear scatterplot: blue circles—plots (means or medians); red solid line—linear correlation line; red dotted lines—0.95 confidence interval.

DR was positively associated with diabetes duration (R = 0.276454, *p* = 0.013), HbA_1_c (R = 0.361563, *p* < 0.001), SBP (R = 0.465979, *p* < 0.001), and DBP (R = 0.379747, *p* < 0.001) (Figure 2A–D). No significant correlation was observed between DR and age, BMI, serum lipids, and renal function (*p* > 0.05).

**Figure 3:**
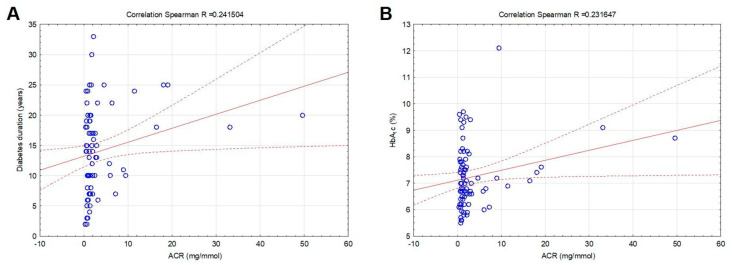
Correlations between the albumin-to-creatinine ratio (ACR ratio), diabetes duration (**A**), and HbA_1_c (**B**) in patients with T2DM included in the study. Legend of the correlation linear scatterplot: blue circles—plots (means or medians); red solid line—linear correlation line; red dotted lines—0.95 confidence interval.

ACR was positively related to diabetes duration (R = 0.241504, *p* = 0.034) and HbA_1_c (R = 0.231647, *p* = 0.043) (Figure 3A,B). No significant relation was observed between the ACR and age, BMI, serum lipids, SBP, and DBP (*p* > 0.05). Estimated GFR was negatively related only to age (R = −0.544649, *p* < 0.001), but no significant relation was found between eGFR and other analyzed variables.

### 3.3. Predictors of Diabetic Retinopathy and Nephropathy

Binary logistic regression analysis (no DR/DR) showed that longer diabetes duration (*p* = 0.008), poor glycemic control (HbA_1_c) (*p* = 0.008), higher SBP (*p* = 0.001), and DBP (*p* = 0.003) were the main predictors of DR. However, even after adjustment for diabetes duration and HbA_1_c, there were significant effects of SBP (AOR = 1.06, *p* = 0.004) and DBP (AOR = 1.12, *p* = 0.007) on DR development (Table 3). No significant relation between DR and other analyzed variables using logistic regression analysis was observed.

The results of backward regression analysis for ACR as a dependent variable are presented in Table 4, and those for eGFR as a dependent variable are in Table 5. The best model for predicting ACR (R^2^ = 0.258) obtained by backward regression included only a longer diabetes duration (*p* = 0.034), while the best model for predicting eGFR (R^2^ = 0.307) involved only age (*p* < 0.001).

## 4. Discussion

The results of our study suggest that SBP and DBP are independent risk factors for DR in patients with T2DM. Less than half of the studied patients with T2DM had DR (46.3%), and most patients had satisfactory glucose control (HbA_1_c 7.1%). Although DR and DN are essential microvascular complications connected with a similar pathogenesis and often coincide, there were no associations between DR and renal function measured with eGFR and ACR. However, the prevalence of DN in our study, defined as an ACR ≥ 30 mg/mmol and estimated GFR < 60 mL/min/1.73 m^2^, was moderate to relatively high (12.5% and 32.6%). Also, no associations existed between DR and age, BMI, and serum lipids. Finally, binary logistic regression analysis (no DR/DR) showed that longer diabetes duration (*p* = 0.008), poor glycemic control (HbA_1_c) (*p* = 0.008), higher SBP (*p* = 0.001), and DBP (*p* = 0.003) were the main risk factors of DR. However, even after adjustment for diabetes duration and HbA_1_c, SBP (AOR = 1.06, *p* = 0.004) and DBP (AOR = 1.12, *p* = 0.007) had significant effects on DR development.

The epidemiological studies confirmed the connection between high blood pressure and DR and showed that blood pressure is not only a risk factor for DR because the treatment of hypertension is beneficial [23]. Large, prospective trials that included patients with T2DM, like ABCD (Appropriate Blood Pressure Control in Diabetes) and UKPDS (The UK Prospective Diabetes Study), suggest that strict blood pressure control can prevent the development and progression of DR [24,25]. Patients with T2DM and SBP ≥ 140 mmHg have a 2.8 times higher risk of developing DR than those with SBP < 125 mmHg [23]. In addition, in those with borderline increased blood pressure (up to 144/82 mmHg), a 35% risk reduction in DR progression and photocoagulation can be achieved with a decrease in SBP by 10 mmHg and with a reduction in DBP by 5 mmHg. In our study, the patients had even lower median systolic and diastolic blood pressure (135/80 mmHg). The Steno 2 study included patients with T2DM and microalbuminuria and found a 55% lower risk of DR progression according to the EURODIAB scale with the intensive control of hypertension [26]. Irrespective of normal or high blood pressure, SBP is significantly related to the DR presence in patients with T2DM, even after adjusting for the duration of diabetes, gender, and HbA_1_c [17]. SBP is also associated with incipient and advanced DR independently of the presence of cardiovascular and renal disease [27]. Besides blood pressure level, its variability is also a risk factor for DR in patients with T2DM [28]. Most studies found a link between DR and SBP, but some researchers discovered that only DBP relates to DR [29]. Contrarily, a randomized controlled ADVANCE trial showed that blood pressure control within the normal range (below 140/80 mm Hg) had no effect in preventing DR progression [30].

Hypertension, pregnancy, autonomic neuropathy, and hyperglycemia increase retinal blood flow and lead to a progression of DR, while normoglycemia and the moderate stenosis of the carotid artery decrease retinal blood flow and have a protective effect on DR [31,32,33,34,35]. Retinal blood flow distribution is regulated by central mechanisms controlled by autonomic innervation and local mechanisms through autoregulation. The impaired autoregulation in patients with T2DM potentiated with hyperglycemia significantly increases blood flow and mean arterial pressure, established a vicious circle between retinal hemodynamic changes, hyperglycemia, and hypertension [32]. Several other conditions are implicated in impaired autoregulation in patients with T2DM, like the reduced contractile capability of retinal pericytes, capillary basement thickening, and arteriolar hyalinosis, resulting in the direct transmission of increased blood pressure to the retinal microvasculature [33]. According to studies, hypertension can also cause oxidative stress and inflammation [36]. It appears that the coexistence of hyperglycemia and hypertension can aggravate inflammation and oxidative stress, both of which are pathogenic processes implicated in the development and progression of DR.

**Figure 4:**
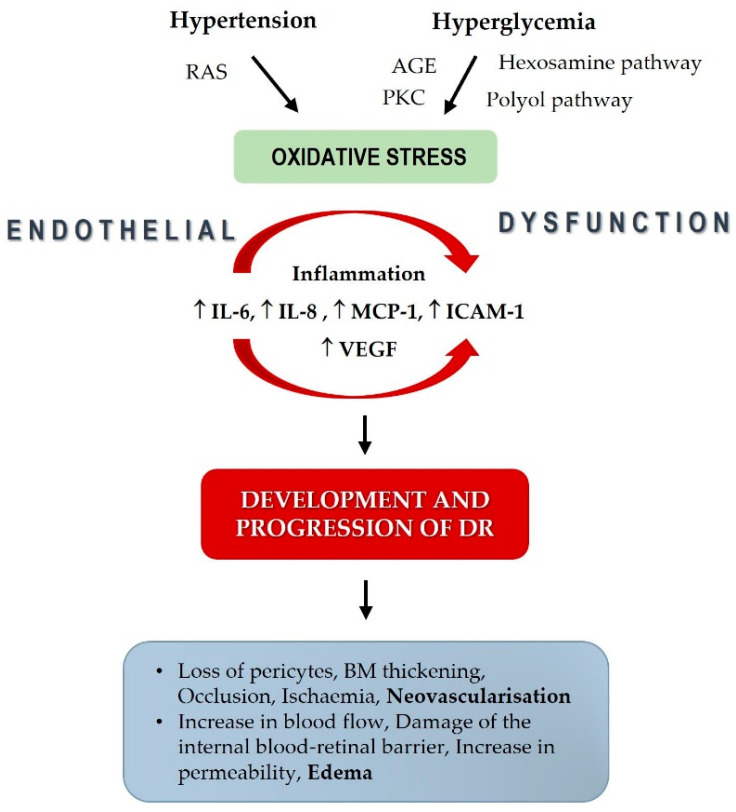
Risk factors for diabetic retinopathy development and progression mentioned in the Discussion.

Increased blood pressure has a role in the pathological changes of DR, participates in the local renin–angiotensin system (RAS), and may damage retinal capillary endothelial cells [27,37]. Although the exact mechanism of high blood pressure damage in patients with DR is not fully understood, it is well known that chronic hyperglycemia via endothelial dysfunction aggravates the blood–retinal barrier, resulting in impaired retinal perfusion [38,39,40]. High blood pressure upregulates vascular endothelial growth factor (VEGF), a potent angiogenic factor and essential growth factor for vascular endothelial cells, which has a vital role in the presence and severity of DR. In addition, an increase in angiotensin-II, observed in patients with hypertension, results in its binding to the AT-I receptor that also upregulates the VEGF [41]. Finally, VEGF induces essential changes related to DR development and progression, like basement membrane thickening, increased vascular permeability, and neovascularization [42] (Figure 4).

Although blood pressure control has protective effects on DR development and progression, the question is which blood pressure value is optimal for patients with DR. It seems that targeting very low systolic and diastolic blood pressure (below 120/80 mmHg) has no additional beneficial effects [43]. A combination of optimal blood pressure control (SBP below 140 mmHg) and glucose control (HbA_1C_ below 7.0%) significantly reduces the probability of DR presence in patients with T2DM [44]. Normotensive patients with T2DM must be screened for DR because about 20% of them have DR, and in those patients, an increase in SBP is independently and significantly associated with DR development and progression [17]. RAS inhibitors should be introduced after diagnosing hypertension in patients with T2DM and DR because of their possible beneficial effects on DR. RAS inhibitors not only decrease the risk of development and progression of DR but also increase the possibility of DR regression, which is suggested in a meta-analysis that included 21 randomized clinical trials with 13,823 participants. Also, ACE inhibitors might be better than angiotensin-receptor blockers in treating DR and are the drug of choice for those with DR [45].

Although the prevalence of DN according to the eGFR category in our study was relatively high (32.6%), DR was not associated with eGFR and ACR. Previous studies observed a significant connection between DR and DN and proposed that DN precedes DR in patients with T2DM [46]. High blood glucose, high systolic and diastolic blood pressure, and duration of disease simultaneously connect the parallel development of DR and DN. However, DR is present and may progress in about 30% of patients with type 1 diabetes and strictly normal renal function (normoalbuminuria and eGFR over 60 mL/min/1.73 m^2^) [19]. In addition, many patients with T2DM and renal abnormalities (proteinuria and/or renal insufficiency) showed no signs of DR [11].

In our study, no significant relation was obtained between the ACR and age, BMI, serum lipids, SBP, and DBP. Although dyslipidemia is related to the pathogenesis of DN, discordant associations of lipid parameters with albuminuria were found [47]. Also, obesity is generally associated with increased ACR, although severe obesity compared with milder obesity status cannot predict the occurrence of increased ACR and microalbuminuria [48]. It is well known that the prevalence of chronic kidney disease and albuminuria rises with age, although recent epidemiological studies suggest that the albuminuria-associated risk of all-cause mortality and cardiovascular mortality is attenuated in elderly patients with diabetes [49]. In addition, the interaction between albuminuria stages and age groups was not significant in elderly individuals of various ages with diabetes [50].

The present study and results have several potential limitations. First, our study was cross-sectional and included a limited number of patients with T2DM. Therefore, the data must be confirmed in prospective studies with more patients. Second, we used a single-office blood pressure measurement that cannot rule out hypertension in patients with diabetes, for which ambulatory blood pressure can provide an excellent prognostic benefit [51]. The procedure used in our study to diagnose the presence and severity of DR and previously described blood-pressure-measurement deficiency may influence the final results, making it difficult to compare findings between studies. Third, since our study included only the white European population, there was no racial/ethnic diversity. Fourth, a small number of DR predictors is also a study limitation.

In conclusion, the results of our study suggest that systolic and diastolic blood pressure are independent risk factors for DR in patients with T2DM with and without DN. The study was conducted on patients with satisfactory glucose control with T2DM. The result of the study implies that we should regularly monitor blood pressure in patients with T2DM, even in those without hypertension and with optimal glucose control, to prevent the development of DR.

## Figures and Tables

**Table 1:** Basic and clinical characteristics, metabolic risk factors, and renal function of all patients with type 2 diabetes (*n* = 160) included in the study.

	All Patients Included in the Study (*n* = 160)
Age (years)	64.3 ± 7.6
Gender (m/f) (%)	60/40
Diabetes duration (years)	14.0 ± 7.1
BMI (kg/m^2^)	29.8 ± 4.7
SBP (mmHg)	135 (110–170)
DBP (mmHg)	80 (70–110)
HbA_1_c (%)	7.1 (5.5–12.1)
Total cholesterol (mmol/L)	4.6 (2.7–10.2)
HDL cholesterol (mmol/L)	1.3 (0.8–2.5)
LDL cholesterol (mmol/L)	2.5 (0.9–7.1)
Triglycerides (mmol/L)	1.6 (0.5–7.0)
Serum creatinine (μmol/L)	75.5 (42–163)
ACR (mg/mmol)	1.4 (0.3–49.6)
eGFR (mL/min/1.73 m^2^)	86.5 (32–108)

Legend: Values are means ± SD, percentages, or medians (min-max). BMI indicates body mass index; SBP: systolic blood pressure; DBP: diastolic blood pressure; HbA_1_c: glycated hemoglobin; HDL: high-density lipoprotein cholesterol; LDL: low-density lipoprotein cholesterol; eGFR: estimated glomerular filtration rate; ACR: albumin-to-creatinine ratio.

**Table 2:** Diabetes duration, systolic and diastolic blood pressure, and HbA_1_c of patients with type 2 diabetes (*n* = 160). Patients were divided into three groups according to the level of diabetic retinopathy.

	No DR (*n* = 86)	Mild/Moderate NPDR (*n* = 44)	Severe NPDR/PDR (*n* = 30)	*p*-Value
Diabetes duration	11.98 ± 6.76	15.60 ± 8.23	16.91 ± 5.88	0.017
SBP (mmHg)	130 (115–170)	137 (110–170)	150 (120–170)	0.001
DBP (mmHg)	80 (70–95)	85 (70–110)	85 (75–100)	0.003
HbA_1_c (%)	6.7 (5.5–9.4)	7.3 (5.8–9.2)	7.8 (5.6–12.1)	0.005

Legend: Values are means ± SD or medians (min-max). SBP indicates systolic blood pressure; DBP: diastolic blood pressure; HbA_1_c: glycated hemoglobin; DR: diabetic retinopathy; NPDR: nonproliferative diabetic retinopathy; NPDR/PDR: nonproliferative diabetic retinopathy/proliferative diabetic retinopathy.

**Table 3:** Results of logistic regression analysis for diabetic retinopathy in patients with type 2 diabetes as a dichotomous dependent variable.

Variable	OR (95% CI)	*p*-Value	AOR (95% CI) *	*p*-Value *
Diabetes duration	1.10 (1.02–1.18)	0.008	/	
HbA_1_c	1.87 (1.17–2.99)	0.008	/	
SBP	1.07 (1.03–1.11)	0.001	1.06 (1.02–1.11)	0.004
DBP	1.12 (1.04–1.21)	0.003	1.12 (1.03–1.22)	0.007

* OR after adjustment for diabetes duration and HbA_1_c. Legend: HbA_1_c indicates glycated hemoglobin; SBP: systolic blood pressure; DBP: diastolic blood pressure.

**Table 4:** Results of backward regression analysis for albumin-to-creatinine ratio in patients with type 2 diabetes as a dependent variable.

	Unstandardized Coefficients		Standardized Coefficients		
Model	B	Std. Error	Beta	t	Sig.
(Constant)	0.138	1.839		0.075	0.941
Diabetes duration	0.252	0.117	0.242	2.155	0.034

Dependent variable: albumin-to-creatinine ratio. R^2^ = 0.258.

**Table 5:** Results of backward regression analysis for estimated glomerular filtration rate in patients with type 2 diabetes as a dependent variable.

	Unstandardized Coefficients		Standardized Coefficients		
Model	B	Std. Error	Beta	t	Sig.
(Constant)	0.138	1.839		0.075	0.941
Age	−1.413	0.247	−0.554	−5.728	<0.001

Dependent variable: estimated glomerular filtration rate. R^2^ = 0.307.

## Data Availability

The data presented in this study are available on request from the corresponding author.

## References

[1] Saeedi P., Petersohn I., Salpea P., Malanda B., Karuranga S., Unwin N., Colagiuri S., Guariguata L., Motala A.A., Ogurtsova K. (2019). Global and regional diabetes prevalence estimates for 2019 and projections for 2030 and 2045: Results from the International Diabetes Federation Diabetes Atlas, 9th edition. Diabetes Res. Clin. Pract..

[2] Tomic D., Shaw J.E., Magliano D.J. (2022). The burden and risks of emerging complications of diabetes mellitus. Nat. Rev. Endocrinol..

[3] Van Hecke M.V., Dekker J.M., Stehouwer C.D., Polak B.C., Fuller J.H., Sjolie A.K., Kofinis A., Rottiers R., Porta M., Chaturvedi N. (2005). EURODIAB prospective complications study. Diabetic retinopathy is associated with mortality and cardiovascular disease incidence: The EURODIAB Prospective Complications Study. Diabetes Care.

[4] Yau J.W., Rogers S.L., Kawasaki R., Lamoureux E.L., Kowalski J.W., Bek T. (2012). Global prevalence and major risk factors of diabetic retinopathy. Diabetes Care.

[5] Klein R., Klein B.E.K. (2002). Blood pressure control and diabetic retinopathy. Br. J. Ophthalmol..

[6] Shaya F.T., Aljawadi M. (2007). Diabetic retinopathy. Clin. Ophthalmol..

[7] Poulsen P.L., Bek T., Ebbehøj E., Hansen K.W., Mogensen C.E. (1998). 24-h ambulatory blood pressure and retinopathy in normoalbuminuric IDDM patients. Diabetologia.

[8] Mbata O., Abo El-Magd N.F., El-Remessy A.B. (2017). Obesity, metabolic syndrome and diabetic retinopathy: Beyond hyperglycemia. World. J. Diabetes.

[9] Grunwald J.E., Alexander J., Ying G.S., Maguire M., Daniel E., Whittock-Martin R., Parker C., McWilliams K., Lo J.C., Go A. (2012). Retinopathy and chronic kidney disease in the Chronic Renal Insufficiency Cohort (CRIC) study. Arch. Ophthalmol..

[10] Arar N.H., Freedman B.I., Adler S.G., Iyengar S.K., Chew E.Y., Davis M.D., Satko S.G., Bowden D.W., Duggirala R., Elston R.C. (2008). Heritability of the severity of diabetic retinopathy: The FIND-eye study. Inv. Ophthalmol. Vis. Sci..

[11] Wolf G., Müller N., Mandecka A., Müller U.A. (2007). Association of diabetic retinopathy and renal function in patients with types 1 and 2 diabetes mellitus. Clin. Nephrol..

[12] Tolonen N., Hietala K., Forsblom C., Harjutsalo V., Makinen V.P., Kyto J., Summanen P.A., Thorn L.M., Wadén J., Gordin D. (2013). Associations and interactions between lipid profiles, retinopathy and nephropathy in patients with type 1 diabetes: The FinnDiane Study. J. Intern. Med..

[13] Li Y., Su X., Ye Q., Guo X., Xu B., Guan T., Chen A. (2021). The predictive value of diabetic retinopathy on subsequent diabetic nephropathy in patients with type 2 diabetes: A systematic review and meta-analysis of prospective studies. Ren. Fail..

[14] Kohner E.M. (1989). Diabetic retinopathy. Br. Med. Bull..

[15] Stratton I.M., Kohner E.M., Aldington S.J., Turner R.C., Holman R.R., Manley S.E., Matthews D.R. (2001). UKPDS 50: Risk factors for incidence and progression of retinopathy in type II diabetes over 6 years from diagnosis. Diabetologia.

[16] Emdin C.A., Rahimi K., Neal B., Callender T., Perkovic V., Patel A. (2015). Blood pressure lowering in type 2 diabetes: A systematic review and meta-analysis. JAMA.

[17] Li Y.T., Wang Y., Hu X.Y., Chen J.H., Li Y.Y., Zhong Q.Y., Cheng H., Mohammed B.H., Liang X.L., Hernandez J. (2021). Association between Systolic Blood Pressure and Diabetic Retinopathy in Both Hypertensive and Normotensive Patients with Type 2 Diabetes: Risk Factors and Healthcare Implications. Healthcare.

[18] Tomić M., Vrabec R., Ljubić S., Bulum T., Prkačin I., Rahelić D. (2021). Renal function is associated with cataract development in patients with type 2 diabetes. Acta. Clin. Croat..

[19] Bulum T., Blaslov K., Duvnjak L. (2014). Risk factors for development and progression of nonproliferative retinopathy in normoalbuminuric patients with type 1 diabetes. Diabetes Res. Clin. Pract..

[20] Levey A.S., Stevens L.A. (2010). Estimating GFR using the CKD Epidemiology Collaboration (CKD-EPI) creatinine equation: More accurate GFR estimates, lower CKD prevalence estimates, and better risk predictions. Am. J. Kidney Dis..

[21] Aldington S.J., Kohner E.M., Meuer S., Klein R., Sjølie A.K. (1995). Methodology for retinal photography and assessment of diabetic retinopathy: The EURODIAB IDDM complications study. Diabetologia.

[22] Wilkinson C.P., Ferris F.L., Klein R.E., Lee P.P., Agardh C.D., Davis M., Dills D., Kampik A., Pararajasegaram R., Verdaguer J.T. (2003). Proposed international clinical diabetic retinopathy and diabetic macular edema disease severity scales. Ophthalmology.

[23] Gillow J.T., Gibson J.M., Dodson P.M. (1999). Hypertension and diabetic retinopathy-what’s the story?. Br. J. Ophthalmol..

[24] Matthews D.R., Stratton I.M., Aldington S., Holman R.R., Kohner E.M., UK Prospective Diabetes Study Group (2004). Risks of progression of retinopathy and vision loss related to tight blood pressure control in type 2 diabetes mellitus (UKPDS 69). Arch. Ophthalmol..

[25] Schrier R.W., Estacio R.O., Mehler P.S., Hiattet W.R. (2007). Appropriate blood pressure control in hypertensive and normotensive type 2 diabetes mellitus: A summary of the ABCD trial. Nat. Clin. Pract. Nephrol..

[26] Gaede P., Vedel P., Parving H., Pedersenet O. (1999). Intensified multifactorial intervention in patients with type 2 diabetes and microalbuminuria: The Steno type 2 randomized study. Lancet.

[27] Liu L., Quang N.D., Banu R., Kumar H., Tham Y.-C., Cheng C.-Y., Wong T.Y., Sabanayagam C. (2020). Hypertension, blood pressure control and diabetic retinopathy in a large population-based study. PLoS ONE.

[28] Lou Q., Chen X., Wang K., Liu H., Zhang Z., Lee Y. (2022). The Impact of Systolic Blood Pressure, Pulse Pressure, and Their Variability on Diabetes Retinopathy among Patients with Type 2 Diabetes. J. Diabetes Res..

[29] Rajalakshmi R., Amutha A., Ranjani H., Ali M.K., Unnikrishnan R., Anjana R.M., Narayan K.M.V., Mohan V. (2014). Prevalence and risk factors for diabetic retinopathy in Asian Indians with young onset type 1 and type 2 diabetes. J. Diabetes Complicat..

[30] Patel A., MacMahon S., Chalmers J., Neal B., Woodward M., Billot L., Harrap S., Poulter N., Marre M., Cooper M. (2007). Effects of a fixed combination of perindopril and indapamide on macrovascular and microvascular outcomes in patients with type 2 diabetes mellitus (the ADVANCE trial): A randomised controlled trial. Lancet.

[31] Kohner E.M., Patel V., Rassam S.M.B. (1995). Role of blood flow and impaired auto-regulation in the pathogenesis of diabetic retinopathy. Diabetes.

[32] Grunwald J.E., Dupont J., Riva C.E. (1996). Retinal haemodynamics in patients with early diabetes mellitus. Br. J. Ophthalmol..

[33] Fuchsjäger-Mayrl G., Polak K., Luksch A., Polska E., Dorner G.T., Rainer G., Eichler H.G., Schmetterer L. (2001). Retinal blood flow and systemic blood pressure in healthy young subjects. Graefes. Arch. Clin. Exp. Ophthalmol..

[34] Pota C.E., Apaydın K.C. Retinal and choroidal microvascular changes during pregnancy detected with OCTA. Can. J. Ophthalmol..

[35] István L., Czakó C., Benyó F., Élő A., Mihály Z., Sótonyi P., Varga A., Nagy Z.Z., Kovács I. (2022). The effect of systemic factors on retinal blood flow in patients with carotid stenosis: An optical coherence tomography angiography study. Geroscience.

[36] Vaziri N.D., Rodriguez-Iturbe B. (2006). Mechanisms of disease: Oxidative stress and inflammation in the pathogenesis of hypertension. Nat. Clin. Pract. Nephrol..

[37] Sjølie A.K., Dodsonand P., Hobbs F.R.R. (2011). Does renin-angiotensin system blockade have a role in preventing diabetic retinopathy? A clinical review. Int. J. Clin. Pract..

[38] Cardoso C.R.L., Leite N.C., Dib E., Salles G.F. (2017). Predictors of development and progression of retinopathy in patients with type 2 diabetes: Importance of blood pressure parameters. Sci. Rep..

[39] Do D.V., Wang X., Vedula S.S., Marrone M., Sleilati G., Hawkins B.S., Frank R.N. (2015). Blood pressure control for diabetic retinopathy. Cochrane. Database. Syst. Rev..

[40] Fraser-Bell S., Symes R., Vaze A. (2017). Hypertensive eye disease: A review. Clin. Exp. Ophthalmol..

[41] Schlingerman R.O., Hinsbergh V.W.M. (1997). Role of vascular permeability factor/vascular endothelial growth factor in eye disease. Br. J. Ophthalmol..

[42] Apte R.S., Chen D.S., Ferrara N. (2019). VEGF in Signaling and Disease: Beyond Discovery and Development. Cell.

[43] Hansson L., Zanchetti A., Carruthers S.G. (1998). Effects of intensive blood pressure lowering and low dose aspirin in patients with hypertension: Principal results of the hypertension optimal treatment (HOT) randomized trial. Lancet.

[44] Pan C.-W., Wang S., Xu C.-L., Song E. (2018). Combined effect of glycemic and blood pressure control on diabetic retinopathy among Chinese with type-2 diabetes mellitus. Diabetol. Metab. Syndr..

[45] Wang B., Wang F., Zhang Y., Zhao S.-H., Zhao W.-J., Yan S.-L., Wang Y.-G. (2015). Effects of RAS inhibitors on diabetic retinopathy: A systematic review and meta-analysis. Lancet Diabetes Endocrinol..

[46] Kotlarsky P., Bolotin A., Dorfman K., Knyazer B., Lifshitz T., Levy J. (2015). Link between retinopathy and nephropathy caused by complications of diabetes mellitus type 2. Int. Ophthalmol..

[47] Sun K., Lin D., Li F., Huang C., Qi Y., Xue S., Tang J., Yang C., Li Y., Ren M. (2015). Discordant associations of lipid parameters with albuminuria and chronic kidney disease: A population-based study. Lipids Health Dis..

[48] Minoo F., Mahdavi-Mazdeh M., Abbasi M.R., Sohrabi S. (2015). Impact of the severity of obesity on microalbuminuria in obese normotensive nondiabetic individuals. J. Renal. Inj. Prev..

[49] Tancredi M., Rosengren A., Svensson A.M., Kosiborod M., Pivodic A., Gudbjörnsdottir S., Wedel H., Clements M., Dahlqvist S., Lind M. (2015). Excess Mortality among Persons with Type 2 Diabetes. N. Engl. J. Med..

[50] Hwang S., Lee K., Park J., Kim D.H., Jeon J., Jang H.R., Hur K.Y., Kim J.H., Huh W., Kim Y.G. (2023). Prognostic significance of albuminuria in elderly of various ages with diabetes. Sci. Rep..

[51] Gupta H., Vidhale T., Pustake M., Gandhi C., Roy T. (2022). Utility of ambulatory blood pressure monitoring in detection of masked hypertension and risk of hypertension mediated organ damage in normotensive patients with type 2 diabetes mellitus. Blood Press..

